# New therapeutic modalities to modulate orthodontic tooth movement

**DOI:** 10.1590/2176-9451.19.6.123-133.sar

**Published:** 2014

**Authors:** Ildeu Andrade, Ana Beatriz dos Santos Sousa, Gabriela Gonçalves da Silva

**Affiliations:** 1 Catholic University of Minas Gerais, School of Dentistry, Adjunct professor, Masters program in Orthodontics, School of Dentistry, Catholic University of Minas Gerais (PUC-MG); 2 Catholic University of Minas Gerais, School of Dentistry, Undergraduate student, School of Dentistry, Catholic University of Minas Gerais (PUC-MG)

**Keywords:** Orthodontic tooth movement, Corticotomy surgery, Gene therapy, Ultrasound

## Abstract

Modulation of orthodontic tooth movement (OTM) is desirable not only to patients because it shortens treatment time, but also to orthodontists, since treatment duration is associated with increased risk of gingival inflammation, decalcification, dental caries, and root resorption. The increased focus on the biological basis of tooth movement has rendered Orthodontics a more comprehensive specialty that incorporates facets of all fields of medicine. Current knowledge raises the possibility of using new therapeutic modalities for modulation of OTM, such as corticotomy, laser therapy, vibration (low-intensity pulsed ultrasound), local injections of biomodulators and gene therapy; with the latter being applicable in the near future. They are intended to enhance or inhibit recruitment, differentiation and/or activation of bone cells, accelerate or reduce OTM, increase stability of orthodontic results, as well as assist with the prevention of root resorption. This article summarizes recent studies on each one of these therapeutic modalities, provides readers with information about how they affect OTM and points out future clinical perspectives.

## Abstract (pt)

A modulação do movimento dentário ortodôntico (MDO) é desejável para os pacientes, pois reduz o tempo de tratamento, e também para ortodontistas, uma vez que a duração do tratamento tem sido associada a um aumento do risco de inflamação gengival, descalcificação, cárie dentária e reabsorção radicular. O crescente foco sobre os mecanismos biológicos da movimentação dentária levou a Ortodontia a ser uma especialidade mais abrangente, que hoje incorpora aspectos de todas as áreas da Medicina. Com o conhecimento atual, o uso de novas modalidades terapêuticas que visam a modulação da MDO, como a corticotomia, terapia a *laser* de baixa intensidade e vibração (ultrassom pulsátil de baixa intensidade) já são uma realidade clínica. Outras, como injeções locais de biomoduladores e a terapia genética, serão utilizadas em breve. Elas destinam-se a aumentar ou inibir o recrutamento, à diferenciação e/ou ativação das células ósseas, a acelerar ou reduzir a MDO, a aumentar a estabilidade dos resultados ortodônticos, bem como auxiliar na prevenção da reabsorção radicular. Esse artigo resume os estudos mais recentes sobre cada uma dessas novas modalidades terapêuticas, fornecendo informações aos leitores a respeito de como afetam a MDO e aponta futuras perspectivas clínicas.

## INTRODUCTION

Research in the field of orthodontic tooth movement (OTM) has evolved rapidly and changed considerably since the work of Reitan et al in the 1950s.^1^ Moreover, the importance of all tissues, be it alveolar bone, periodontal ligament (PDL), root cementum, and associated vascular and neural networks, has been investigated to delineate the role played by them.^2^ This growing attention given to the biological basis of Orthodontics expands current knowledge and augments understanding of the effects produced by mechanical loading over living tissues. Orthodontics, which for a long time was considered a technique-oriented profession, has steadily evolved to a comprehensive specialty that incorporates aspects of all fields of medicine, emphasizing that live human beings are being treated instead of dental typodonts, only. Moreover, a sound biological background is critical for the well-educated clinician to ensure optimal evidence-based treatment plan and to promote clinical excellence.

OTM is a biological process characterized by PDL and alveolar bone remodeling in response to an orthodontic force which will promote extensive cellular and molecular changes in the periodontium. Orthodontic treatment time ranges between 21-27 and 25-35 months for nonextraction and extraction therapies, respectively.^3^^,^ 4

Accelerating the rate of tooth movement is desirable to orthodontists because treatment duration has been associated with an increased risk of gingival inflammation, decalcification,^5^ dental caries, and, especially, root resorption.^6^ Shorter treatment duration with consequent lower costs are also important to all patients, particularly to adults who have been increasingly seeking treatment.^7^ However, adult patients typically require longer treatment time due to having slower metabolism in comparison to younger patients.^8^

It has been estimated that teeth move 0.8-1.2 mm/month when continuous forces are applied.^9^ Since the best way to shorten treatment time is to speed up tooth movement, new therapeutic modalities have been reported to this end.^10^ Tooth movement has been accelerated by local injection of biomodulators, application of laser therapy, mechanical vibration and gene therapy, as well as by corticotomies. Some of these approaches cannot yet be applied clinically; but others, such as corticotomy, laser therapy and vibration are somewhat already part of the therapeutic arsenal. Nevertheless, a question remains. How can these procedures accelerate or inhibit tooth movement? Since OTM is a biological process, any procedure used to modulate OTM is direct or indirectly related to the cellular and molecular mechanisms involved in the biology of tooth movement. The aim of the present review is to summarize recent studies on each of these therapeutic modalities and to provide readers with information about how they affect OTM. 

## BIOLOGY OF TOOTH MOVEMENT

Orthodontists work in a unique biological environment wherein applied forces engender remodeling of both mineralized (alveolar bone) and nonmineralized (PDL and gingiva) paradental tissues, including associated blood vessels and neural elements. Bone remodeling processes begin when an orthodontic force is applied over the periodontium which, in turn, generates aseptic inflammatory response. This inflammation alters homeostasis and microcirculation of PDL, thereby creating areas of ischemia and vasodilatation, which results in the release of several biological mediators, such as cytokines, chemokines, growth factors, neurotransmitters, metabolites of arachidonic acid and hormones. These molecules trigger a number of cellular responses that will promote bone resorption by osteoclasts in the pressure sites and bone formation by osteoblasts in the tension sites.^2^

Osteoclasts are multinucleated cells derived from precursors in the myeloid/monocyte lineage that circulate in the blood after being formed in the bone marrow. They are the only cells in nature that can degrade mineralized bone tissue and are important for physiological remodeling and modeling processes, calcium homeostasis, tooth eruption, and OTM. Mature osteoclasts attach to bone surface by a sealing zone. In this area, proton pumps and chloride channels are expressed. They are important for extracellular acidification and demineralization of bone. Proteolytic enzymes are then released and degrade the extracellular matrix proteins.^11^ Therefore, when alveolar bone is stimulated to resorb by means of an orthodontic force, a sequence of events is initiated and ultimately result in recruitment, differentiation, activation and maintenance of osteoclasts in bone remodeling sites. Osteoclastogenesis begins with stem cell division and proliferation of osteoclast precursors cells in hematopoietic tissues (bone marrow, spleen, liver and peripheral blood). The second step is the migration of cells to bone resorption sites where they will be differentiated and activated. Tooth movement efficiency is directly linked, quantitatively and qualitatively, to recruitment, differentiation, activation and maintenance of these cells in these sites.^12^

Since osteoclasts are bone specific cells, they are recruited from blood stream by chemotactic factors released from components of bone matrix and osteoblasts.^2^ After proliferation and migration of osteoclast precursors to bone remodeling sites, these progenitors will differentiate when their receptor c-Fms interacts with the ligand macrophage colony-stimulating factor (M-CSF), which is also important for osteoclast survival. Specific differentiation of osteoclasts is due to activation of RANK (receptor activator of nuclear factor-kB) by RANKL (receptor activator of nuclear factor-kB ligand) expressed by stromal cells in bone marrow and osteoblasts.^12^

Osteoblasts are of mesenchymal origin and are responsible for bone formation during embryonic development, growth, bone remodeling and fracture healing. In Orthodontics, bone formation begins 40-48 hours after force application in PDL tension sites.^2^ Osteocytes, which are osteoblasts that become embedded in their own bone matrix, participate in the process of osteogenesis, being acutely sensitive and responsive to applied tensile orthodontic forces. Their cellular projections favor communication with neighboring osteocytes, as well as with alveolar bone surface-lining cells and bone marrow cavity cells. Osteoblasts, which maintain direct contact with osteocytes, respond to these signals and initiate bone apposition.^13^ Moreover, stretched PDL fiber bundles stimulate cell replication.^2^ Stem-cells (pericytes) which migrate from blood vessel walls, and mesenchymal stem-cells differentiate into pre osteoblastic cells 10 hours after force application.^2^ Chemokines, cytokines, and growth factors are directly involved in this process.^13^^,^ 14 Osteoblasts also positively regulate osteoclast activity by expressing cytokines such as RANKL, a key activator of osteoclast differentiation, and negatively by expression of osteoprotegerin (OPG), a soluble decoy receptor that inhibits RANKL.^12^^,^ 13 Other cytokines playing a role in bone remodeling induced by orthodontic forces are: tumor necrosis factor (TNF)-α, interleukin (IL)-1β, IL-2, IL-3, IL-4, IL-5, IL-6, IL-10, interferon-γ (IFN-y), tissue biomarkers (matrix metalloproteinases (MMP)-1, MMP-2, MMP-9, tissue inhibitors of MMPs (TIMP)-1 and 2), and chemokines (CCL2, CCL3, CCL5, CCL7, CCL9, CXCL-8, CXCL9, CXCL10, CXCL12 and CXCL-13), all of which play a central role in trafficking and homing of leukocytes, immune cells and stromal cells.^13^^,^ 15 Mechanical loading also stimulates local expression of many growth factors (GFs) (i.e: vascular endothelial GF (VEGF), transforming GF (TGF)-β, bone morphogenetic proteins (BMPs), insulin-like GF (IGFs) and fibroblast GF (FGF) involved in bone and PDL remodeling in the early stages of OTM in both tensile and compressive sites.^13^

Taken all together, chemokines, cytokines and growth factors (Gfs) are the main molecules involved in bone cell recruitment, activation, proliferation, differentiation and survival. These molecules stimulate PDL and bone cells to orchestrate an inflammatory response followed by osteoclastogenesis and bone resorption in compression sites, and bone neoformation by osteoblasts at PDL tension sites. Research trend is now directed toward elucidating the molecular mechanisms involved in the aforementioned events. Current knowledge raises the possibility of using therapeutic modalities (local injections of biomodulators, laser therapy, mechanical vibration, gene therapy, and corticotomy) capable of acting on or increasing the expression of specific cytokines, chemokines and GFs. These molecules can modulate the outcomes of orthodontic force application, accelerating OTM, enhancing biological anchorage at specific sites, possibly decreasing the rebound effect, and assisting with the prevention of root resorption.

## CORTICOTOMY

**Figure 1.**
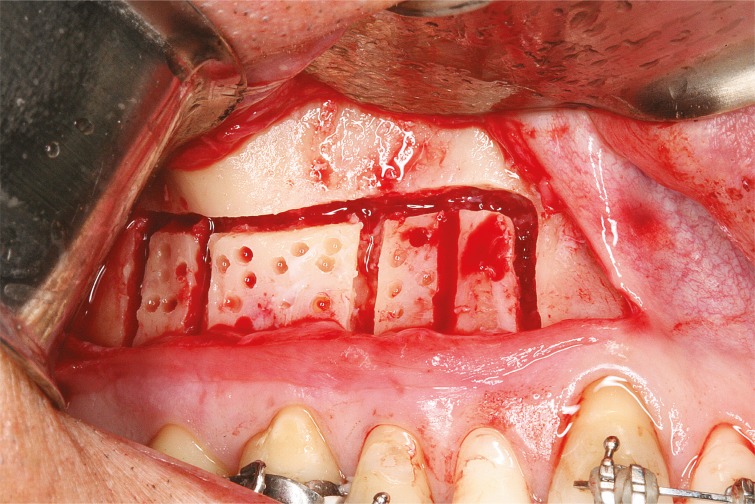
Corticotomy. Mucoperiosteal flaps are raised and corticotomy carried out on buccal and palatal surfaces. Monocortical perforations are performed in areas of intended tooth movement.

Over the past 10 years, corticotomies have become a popular means of increasing the rate of tooth movement. In corticotomy, the cortical layer is cut or perforated to the depth of the medullary bone which is preserved (Fig 1). During bone healing process, a regional acceleratory phenomenon (RAP) takes place in the periodontium. RAP is a natural localized reaction of soft and hard tissues in response to an injury, and is associated with increased perfusion, bone turnover and decreased bone density.^16^^,^ 17 It is similar to the processes associated with normal fracture healing which include a reactive phase, a reparative phase, and a remodeling phase. The reactive phase lasts 7 days and it is characterized by immediate constriction of blood vessels to mitigate bleeding, followed by hematoma within a few hours.^16^ The cells within the hematoma will die and a loose aggregate of fibroblasts, intercellular materials and other supporting cells is then formed. This granulation tissue is formed within approximately two weeks.^18^ A few days later, periosteal cells surrounding the injury site and the granulation-tissue fibroblasts will be transformed into chondroblasts and form hyaline cartilage. Periosteal cells distal to the injury site develop into osteoblasts which form woven bone.^19^ The association of the mass of hyaline cartilage and woven bone is called callus and will be replaced by lamellar bone in the subsequent phase. In fractures, the time between callus formation and mineralization is of 1-4 months;^16^ corticotomies are expected to heal faster than fractures (2-3 months). The last phase of healing takes 1-4 years and it is characterized by complete remodeling of the bone into functionally mature lamellar bone.

Tooth movement should be faster in less dense alveolar bone which is rapidly remodeled for the same reasons tooth movement is faster in growing children than in adults.^20^ Moreover, animal studies showed that corticotomies provide three times as many osteoclasts, three times greater bone apposition rate and a twofold decrease in calcified trabecular bone.^20^ Moreover, another study demonstrated that perforations in the cortical bone increase the expression of 37 inflammatory cytokines, which leads to more osteoclasts and, consequently, greater bone remodeling process.^21^

Although effective and highly predictable, corticotomy-assisted orthodontic treatment is quite invasive as it requires extensive flap elevation and bone surgery. A previous study proposed the use of a piezoelectric knife instead of a high-speed surgical bur to decrease surgical trauma and still achieve rapid tooth movement. Due to its micrometric and selective cut, piezoelectric devices have been claimed to produce safe and precise osteotomies without osteonecrotic damage.^22^

Taken all together, there is twice as much tooth movement with than without corticotomies. However, this window of opportunity used to accelerate tooth movement is limited to 2-3 months, in which 4-6 mm of tooth movement might be expected (twice as much the normal rate).^20^ Nevertheless, further controlled clinical trials are needed to determine the actual effects of corticotomies.

## LASER THERAPY

**Figure 2.**
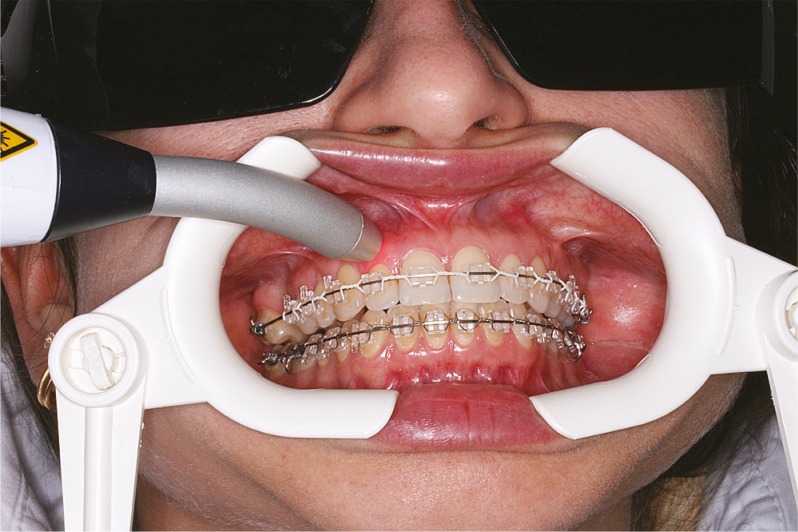
Laser irradiation. Application of LLLT in areas of intended tooth movement.

The term "laser" originated as an acronym for "light amplification by stimulated emission of radiation". It is a device that emits light through a process of optical amplification based on the stimulated emission of electromagnetic radiation.^23^ Lasers differ from other light sources by their coherence which allows them to be focused to a limited spot, to stay narrow over long distances or to have a very narrow spectrum (emitting a single color of light). In medicine, lasers have many important applications: bloodless surgery, laser healing, surgical treatment, kidney stone treatment, eye treatment and many others. The laser technique has also been widely applied in Dentistry; in orthodontic treatment, it has proved to have many benefits. They can be used to perform gingivectomy, frenectomy, surgical exposure of tooth (with less bleeding and swelling, improved precision, reduced pain and improved healing), enamel etching, bonding, bracket debonding, pain control, treatment of traumatic ulcers in the oral mucosa and to accelerate tooth movement^24^^,^ 25 (Fig 2). 

Lasers can be classified as low and high-intensity lasers of which main differences are their potency and mechanism of action.^42^ High-intensity lasers, such as the CO_2_ laser, Nd laser: Yttrium aluminum garnet (Nd:YAG), argon laser, Er:YAG laser, and the excimer laser act by increasing the temperature, showing a destructive potential, and are usually used in surgical procedures. Meanwhile, the low-intensity laser (also known as soft laser, cold laser or laser therapy) does not have a destructive potential. Its photobiomodulation mechanism of action penetrates tissues and stimulates cellular metabolism, bone remodeling and tooth movement which is of greatest interest in Orthodontics.^24^^,^ 25 Different low-energy laser modalities have been used in different doses and in various treatment protocols, including helium-neon (632.8 nm wavelength) and semiconductor lasers (emitting light in the range of 780-950 nm), gallium-aluminum-arsenide (GaAlAs) (805 ± 25 nm wavelength) and gallium-arsenide (904 nm wavelength).^26^ GaAlAs diode laser has been repeatedly used in the past years and has proved to have higher depth of tissue penetration in comparison to other modalities, therefore, providing the clinicians with a suitable penetrative instrument with great efficiency in orthodontic treatment.^27^

The exact mechanism of laser-cell interaction is still to be investigated. The stimulation of photoreceptors in the mitochondrial respiratory chain, changes in cellular ATP levels and cell membrane stabilization have been discussed.^28^ It is generally accepted that laser effects on cells are wavelength and dose-dependent. The existence of a "window of specificity" at certain wavelengths and energy dosages has been postulated.^29^ Molecular absorption of laser light is a prerequisite for any cellular effect.

A previous study^30^ demonstrated that low-level laser therapy (LLLT) stimulates cellular proliferation and differentiation of osteoblast lineage nodule-forming cells, especially in committed precursors, resulting in an increase in the number of differentiated osteoblastic cells as well as in bone formation. Meanwhile, another study found that low-energy laser irradiation stimulated the amount of tooth movement and formation of osteoclasts on the side of pressure during experimental tooth movement *in vivo*. As bone remodeling is a physiological process that involves osteoclastic bone resorption and osteoblastic bone formation,^2^ those findings are not surprising. Furthermore, recent studies showed that low-energy laser irradiation accelerated orthodontic movement of human teeth.^31^^,^ 32

However, the effect of LLLT on tooth movement is reportedly controversial, as different stimulatory, inhibitory and irrelevant effects have been shown in the literature. A previous study^33^ reported that low-energy laser irradiation significantly inhibited the production of prostaglandin E (PGE2), and that interleukin (IL-1β) was increased by mechanical stress *in vitro*. If low-energy laser irradiation functions to inhibit these pro-inflammatory cytokines, OTM might be slow. Another LLLT study^34^ demonstrated low stimulatory or inhibitory effect on the rate of orthodontic tooth movement. Conversely, other studies^35^^,^ 36 reported that IL-1, RANKL, M-CSF, MMP-9, cathepsin K, and α(v)β3 integrin were stimulated via their respective pathways during the differentiation of bone cells, and the amount of tooth movement was increased by low-energy laser irradiation.^37^ Moreover, an *in vitro* study^35^ showed that the gene expression of RANK in osteoclast precursor cells increased when cells were irradiated with low-energy laser. On the basis of the findings of this review, it is possible to assert that LLLT speed up tooth movement via RANK ⁄ RANKL expression.

Although further studies are necessary to evaluate the effects of different irradiation dosages, the prolonged use of laser irradiation on tooth movement or bone remodeling, or both, and the introduction of laser therapy at an early stage of tooth movement in orthodontic treatment seem feasible and may be of great therapeutic benefit to abbreviate treatment time.

## VIBRATION

Tooth movement is closely related to response to applied orthodontic forces that cause remodeling of periodontal tissues, especially the alveolar bone. Bone is a highly specialized form of connective tissue and consists of a cortical bone that overlies the softer inner structure named cancellous or trabecular bone. Its formation and regeneration involve chemotaxis, cell proliferation, differentiation and synthesis of extracellular matrix; a result of interaction established amongst biochemical, biomechanical, cellular and hormonal signals.^2^

Low-intensity pulsed ultrasound (LIPUS) stimulation is a clinically established, widely used and FDA (Food and Drug Administration) approved intervention for accelerating bone growth during healing of fractures, non-unions and other osseous defects. Therapeutic ultrasound is also widely used, especially in sports medicine and myofunctional therapy, to decrease joint stiffness, reduce pain and muscle spasms, and improve muscle mobility^38^. The frequency and intensity of ultrasound used not only for imaging the human brain (7.5-20 MHz), but also for operative procedures (1 to 3 W/cm^2)^ are much higher than that used for LIPUS which generally uses frequencies varying between 0.5 - 1.5 MHz frequency pulses (with a pulse width of 200 µs) and intensity output of 30 mW/cm^2^ (which is the output signal of devices approved for clinical use), 5-20 minutes per day.^39^^,^ 40

LIPUS is a form of physical energy that can be delivered into living tissues as acoustic intensity waves. *In vivo* 41 and *in vitro* 42 ^,^ 43 studies have shown the direct effect of LIPUS on bone cells.

Although the mechanism by which LIPUS increases the rate of fracture healing is unclear, it is known that the mechanical strains received by cells are translated into biochemical events.^44^ LIPUS, in essence a wave of alternating pressure, is translated into an extracellular mechanical force at the cell membrane where it is transduced into intracellular electrical and/or biochemical signals. Previous studies indicate that LIPUS accelerates the differentiation pathway of mesenchymal stem cells in the osteogenic lineage via activated phosphorylation of MAPK (mitogen-activated protein kinase) pathways,^45^ up-regulation of cyclo-oxygenase-2 (COX-2), prostaglandin E2 (PGE2),^40^ altering the OPG/RANKL ratio in the microenvironment.^42^ and stimulating the production of bone morphogenetic proteins.^43^

As bone, the PDL is also a dynamic tissue which is constantly being remodelled to adapt to mechanical loading. Therefore, it is expected that an appropriate level of mechanical stress be able to induce an anabolic response of the periodontium. The PDL is both the medium of force transfer and the means by which alveolar bone remodels itself in response to applied forces. Moreover, PDL cells (PDLCs) play an important role not only in the maintenance of the periodontium, but also in promoting periodontal regeneration during and after the OTM.^2^ They are a heterogeneous cell population, including cells at different stages of differentiation and lineage commitment. Mechanical vibration can affect osteogenesis by increasing the commitment of PDLSCs to the osteogenic lineage. A previous study has shown that the protein levels of RUNX2 and OSX (transcription factors that play a role in the differentiation and activation of osteoblasts) were both prominently enhanced under ultrasound stimulation.^46^

**Figure 3.**
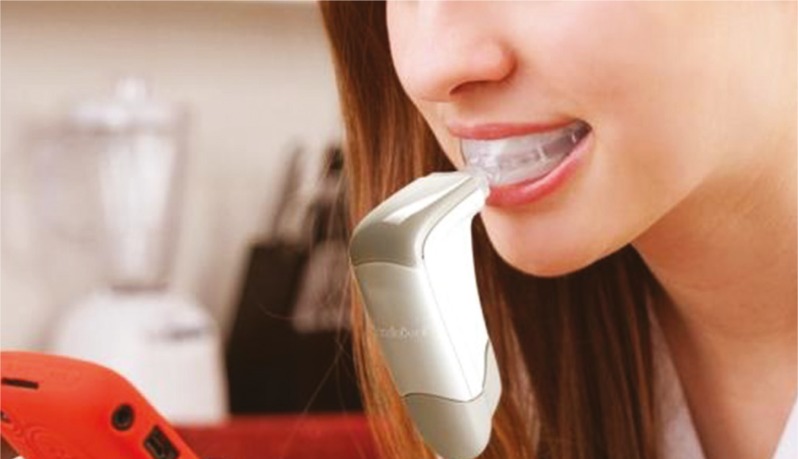
Low-intensity pulsed ultrasound. LIPUS stimulation used to accelerate OTM (Acceledent, Ortho Accel Techonologies, Huston, USA).

It has also been shown that LIPUS (Fig 3) stimulation accelerates OTM by increasing osteoclast number and activity, probably by enhancing the expression of RANKL on the pressure sites.^47^^,^ 48 These same studies have hypothesized that resonance vibration might prevent blood flow obstruction and hyalinization at the compression sites. Furthermore, LIPUS minimizes orthodontically induced tooth root resorption by enhancing dentine and cementum deposition, thereby forming a preventive layer against root resorption.^49^

In short, LIPUS has many clinical advantages, including the fact that it is a biological stimulus, easy to use and noninvasive, in addition to being widely used in clinical medicine.

## LOCAL INJECTION OF BIOMODULATORS

**Figure 4.**
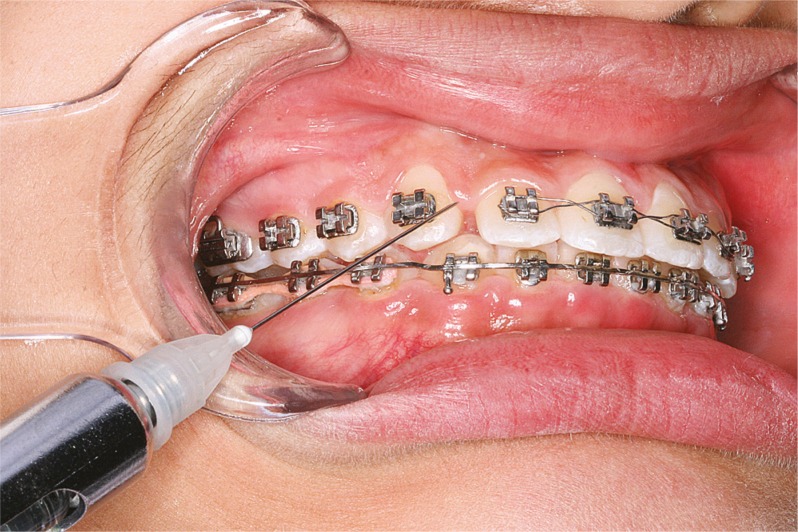
Injection of biomodulators. Injection of inflammatory mediators in the periodontium.

Orthodontic forces create areas of tension and compression in the PDL, which affects remodelling of the periodontium. Following mechanical stress, changes to vascularity and blood flow within the PDL are induced by signalling molecules. The signalling cascade initiates with arachadonic acid metabolites (eicosanoids), neurotransmitters, (substance P and calcitonin gene-related peptide) and second messengers, such as cyclic AMP, phosphoinositol phosphate and diacyl-glycerol.^2^ These molecules trigger the release of cytokines, growth factors and colony stimulating factors, which affect biological mediators such as RANKL, OPG, MMPs and TIMPs.^13^ Recent research advances have suggested that these biological modulators, which enhance or inhibit recruitment, differentiation or activation of osteoclasts, could be used to provide new adjunctive approaches to orthodontic treatment. In other words, local injections of biomodulators could be used to accelerate OTM, reduce root resorption, enhance anchorage and improve stability of orthodontic results (Fig 4).

Numerous reports have described the pharmacological acceleration of OTM through activation of osteoclasts. A previous study^50^ reported that vitamin D3 activated osteoclasts and accelerated OTM. Local administration of prostaglandins (PGs),^51^ osteocalcin,^52^ or PTH^53^ also induced OTM. However, because these drugs are rapidly flushed by blood flow, daily systemic administration or daily local injection are needed. In addition, frequent injections of this substances in local regions may evoke fear in patients and cause problems in medical treatment.

The undesired movement of anchor teeth and the relapse of previously moved teeth are major clinical problems in Orthodontics. Recent research advances suggest that biological modulators which inhibit osteoclasts could be used to address these problems and provide new adjunctive approaches to orthodontic treatment. Several inhibitors have been examined, including bisphosphonates and osteoprotegerin (OPG), and their efficiency in preventing tooth movement has been proved in animal models.^54^^,^ 55

Moreover, advances in understanding cytokine-mediated development and progression of rheumatoid arthritis have led to efforts to neutralize these cytokines by using antibody or soluble receptor techniques.^56^ Soluble receptors are able to bind their ligands with specificity and affinity, and effectively neutralize cytokine activity.^57^ It has been shown in animal models that systemic application of soluble receptors to IL-1 (sIL-RII) or TNF-α (sTNF-α-RI) leads to reduction or even prevention of root resorption.^57^ The concentration of these soluble receptors in the local microenvirement of the target periodontium was also sufficient to interfere in the remodeling processes induced in the periodontal tissues, reducing the number of osteoclasts and, consequently, the amount of OTM.^58^

Nevertheless, routine clinical use of these biomodulators in orthodontics still requires further investigations, to determine the correct dosage, frequency of administration and, especially, the possible local and systemic side effects of its long term use.

## GENE THERAPY

**Figure 5.**
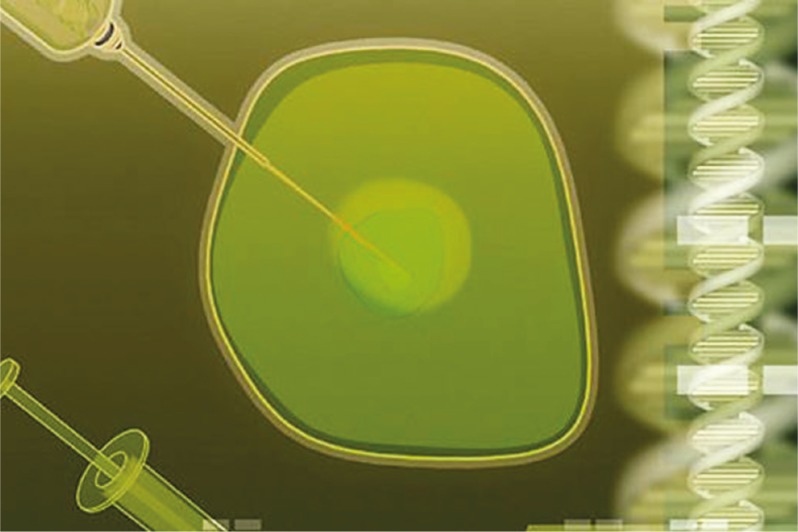
Gene therapy. Delivering a gene to a given cell allows the inserted gene product to be expressed constitutively

The original premise behind gene therapy (GT) in the 90s was the believe that if a defective gene resulting in a specific disease could be replaced by a healthy gene, then the disease could be cured.^59^ However, the potential role of GT as a clinical tool has expanded and it is no longer limited to replacement of defective genes, but rather has become a tool for producing individual proteins to specific tissues and cells (Fig 5). Although all cells contain the genes for all proteins, cells derived from a particular tissue express only a limited selection of these proteins. With GT, it is possible to deliver a gene to a given cell, which allows the inserted gene product to be expressed constitutively.

Modern technology has allowed the manufacture of these proteins (human recombinant proteins) for therapeutic use. However, their life spam is short after injection into the human body. As GT provides the gene for protein production rather than just replacing degradable protein, it achieves higher and more constant levels of protein expression. For this reason, it has became an effective method used to deliver these proteins to specific tissues.^60^

Once protein and location of protein delivery have been chosen, the next step is to choose the vector to deliver the protein. The objective is to get the DNA that encodes the specific protein into the target cell and force it to express the desired protein. The most common delivery vector is by means of a virus, a process also known as "transduction." Nonviral vectors are also used, in which case the process is referred to as "transfection". It is carried out by means of several methods, including liposome and gene gun.^61^ The easiest way to implement local GT is by injecting the vector into a specific tissue. The vector may be delivered systemically to all cells in the body (as in treatment of metastatic diseases) or locally to the target tissue, only (as desired in Orthodontics). Direct GT has been effectively used in knee and ankle joints, skeletal muscle, bone and ligaments.^61^^,^ 62 Nevertheless, in indirect GT, target cells are harvested from the patient and then reinserted. It is advantageous for being able to accurately select a particular cell as the protein delivery vehicle. The indirect method has been effectively used to target articular cartilage, spine and human metacarpophalangeal joints.^61^

Numerous reports have described the pharmacological acceleration of OTM through activation of osteoclasts. However, due to their rapid flush out by blood circulation, daily systemic administration or daily local injection is needed. Local gene transfer has two advantages.^63^ First, it maintains local effective concentration and prolonged protein expression, regardless of blood circulation. Second, protein expression occurs at a local site, thereby avoiding systemic effects.

A previous animal study demonstrated that transfer of RANKL gene to periodontal tissue activated osteoclastogenesis and accelerated OTM without producing any systemic effects.^64^ When comparing corticotomy surgery and RANKL gene transfer to periodontal tissue as two methods that might substantially reduce orthodontic treatment time, RANKL GT demonstrated higher efficacy than standard surgical methods.^65^ Local GT has also been used to inhibit OTM, which might be, in the near future, an important tool to enforce the anchorage unit or increase stability of orthodontic results. Local OPG gene transfer significantly inhibited RANKL-mediated osteoclastogenesis in the periodontium caused by experimental tooth movement.^66^ Moreover, local OPG gene transfer might be a biologic method employed to prevent or inhibit relapse after orthodontic treatment.^67^ Other local or systemic pharmacological agents, such as bisphosphonates and simvastatin, also decrease the extent of initial relapse, but they are rapidly distributed by blood circulation and, for this reason, require daily systemic administration.

Local OPG gene transfer is also clinically relevant for enhancing external root resorption (ERR) repair during retention.^68^ However, the precise biological mechanism behind this finding has not yet been fully elucidated and further studies are required to assess the role of RANK ⁄ RANKL ⁄ OPG axis in ERR repair.

In short, GT is a pioneering new therapeutic modality based on complex biological systems occurring at the leading edge of biomedical knowledge. It offers an alternative method to deliver proteins to a given target tissue, which, in turn, can enhance or inhibit osteoclast recruitment and lead to a more or less OTM. Nonetheless, further research is needed to determine the safety and efficacy of these techniques.

## CONCLUSION

Understanding the biology of tooth movement and treatment outcomes individually is a complex process that requires knowledge in many different areas of biomedicine. The rapid development of molecular biology along with translational studies in humans and experimental systems are likely to provide us with a much more thorough insight into the cellular and molecular mechanisms involved in the bone remodeling processes induced by orthodontic forces. This is a prerequisite to understand the responses in different individuals and to develop new mechanisms by which tooth movement could be regulated not only by mechanical forces, but also by biological agents, if needed.

Basic researchers continue, at an increasing pace, to contribute to the advancement of clinical Orthodontics. Publications on the outcomes of well-planned investigations in every field of medicine inspire researchers who have selected the areas that may be helpful in addressing orthodontic clinical issues faced by the clinician on a daily basis. The biological uniqueness of each patient dictates the need for continuous acquisition of knowledge. Current researches tend to focus on areas such as monitoring patient's reaction to mechanical forces by searching bone remodeling markers in the GCF, saliva, and blood serum. Special attention is given to the speed of tooth movement enhanced by adding certain physical and chemical agents to mechanical orthodontic force. Moreover, current knowledge raises the possibility of enhancing biological anchorage at specific sites, thereby decreasing the rebound effect and assisting with prevention of root resorption.

These new therapeutic modalities have yielded major accomplishments, but new challenges have arisen, which requires continuous investigative efforts in both the research laboratory and the associated clinic.
